# Polymer Composite Analysis and Characterization

**DOI:** 10.3390/polym15081812

**Published:** 2023-04-07

**Authors:** Francesc X. Espinach, Quim Tarrés

**Affiliations:** 1 LEPAMAP-PRODIS Research Group, University of Girona, C/Maria Aurèlia Capmany 61, 17003 Girona, Spain; 2 Serra Hunter Programme Fellowship, Generalitat de Catalunya, Barcelona, Spain

This Special Issue aimed to provide a forum for the discussion of recent advances in the analysis and characterization of polymer-based composites. Authors were encouraged to submit contributions dealing with composites based on oil-derived polymers and biopolymers, as well as natural or mineral fibers as reinforcement. Studies devoted to nanocomposites were also welcomed. The scope of this Special Issue is from basic research on the chemical structure of the composites and their interfaces to applied research on the mechanical and micromechanical properties of the materials. The issue also includes studies on the life cycle assessment or environmental impact of the composites, as well as the possible uses of such materials. In brief, the scientific papers published in this thematic issue on “Polymer Composite Analysis and Characterization” were devoted to the following aspects.

The study of flame retardants to eliminate halogen-based materials is of great importance in the development of new sustainable materials. In this regard, Yuanzhao Zhu et al. published the paper entitled “Morphology-Controlled Synthesis of Polyphosphazene-Based Micro- and Nano-Materials and Their Application as Flame Retardants” [1]. In particular, they prepared poly(cyclotriphosphene-co-4,4′-sulfonyldiphenol) (PZS) microspheres, nanotubes, capsule-type nanotubes, and branched nanotubes as flame retardants. They observed how the increase in reaction temperature affects the morphology of nanotubes to microspheres. The authors determined that the morphology has a remarkable influence on the behavior of these materials as flame retardants, with the nanotubes performing the best among the investigated systems. Additionally, the formation of nanotube capsules with CO2 release was also evaluated. The authors presented these new flame retardants as being viable and more environmentally friendly alternatives to existing materials. In addition, with the development of flame-retardant materials, Diego Pugliese and Giulio Malucelli published the paper “UV-LED Curable Acrylic Films Containing Phosphate Glass Powder: Effect of the Filler Loading on the Thermal, Optical, Mechanical, and Flame Retardant Properties” [2]. The authors evaluated the effects of the incorporation of a micrometer phosphate glass powder on the morphology, as well as the thermal, optical, mechanical, and flame-retardant properties of UV-LED curable acrylic films. The authors determined a formulation that would allow the use of these films as flame-retardant coatings. Their formulation includes the presence of phosphate glass powder which slightly increased the glass transition temperature of the acrylic network, and improved the thermal and thermo-oxidative stability of the cured products.

Furthermore, Zhuoran Zhang et al. presented the paper “Thermal Stability and Flammability Studies of MXene-Organic Hybrid Polystyrene Nanocomposites” [3]. In this work, they evaluated the application of a 2D hybrid MXene–organic (O-Ti_3_C_2_) nanomaterial as a polystyrene nanofiller. The resulting polystyrene nanocomposite (PS/O-Ti_3_C_2_) showed good thermal stability and low flammability. The authors concluded that the thermal stability, 2D thermal barrier, and mass transfer effect of the hybrid MXene–organic nanosheets play an essential role in delaying polymer degradation.

Claudia Forte et al. published the work “The Thermo-Oxidative Behavior of Cotton Coated with an Intumescent Flame Retardant Glycine-Derived Polyamidoamine: A Multi-Technique Study”, where linear polyamidoamines (PAAs) derived from the polyaddition of natural α-amino acids and N,N′-bis(acrylamide) methylene act as intumescent flame retardants for cotton [4].

A fifth article on flame-retardant materials was included in this Special Issue. The paper by Sitong Zhang et al. entitled “The Influence of Fly Ash on the Foaming Behavior and Flame Retardancy of Polyurethane Grouting Materials” [5] studied the improvement in flame-retardant properties in polyurethane (PU) grouting material. The authors used fly ash (FA) as surface-modified polyurethane (PU/FA) fillers. The effects of this filler on the structure, foaming behavior, thermal stability, mechanical properties, hydrophobic properties, and flammability of the obtained materials were examined.

Angxuan Wu et al. used this same material (fly ash) in the paper “Preparation and Finite Element Analysis of Fly Ash/HDPE Composites for Large Diameter Bellows” [6] to produce high-density polyethylene composites. In this work, the authors evaluated the improvement in the mechanical properties of the bellows, using fly ash surface-treated with silane as a reinforcement for high-density polyethylene (HDPE) to develop composite materials. For the compatibilization of both materials, HDPE-g-maleic anhydride was used and the extrusion mixing and injection molding processes were carried out, obtaining significant improvements in the mechanical properties of the material.

Also, in the field of composite materials, Serra-Pararareda et al. presented the paper “Effective Young’s Modulus Estimation of Natural Fibers through Micromechanical Models: The Case of Henequen Fibers Reinforced-PP Composites” [7]. In this study, the properties of polypropylene composites reinforced with henequen fibers were evaluated. Young’s modulus of the fibers was estimated through micromechanical modeling and was further corroborated by a single-strand tensile test after applying a correction method. The authors showed how sisal fibers present a stiffening capacity of PP composites comparable to that of glass fibers. They determined the great potential of sisal fibers as the reinforcement of PP, increasing the sustainability of such composites. Similarly, in another interesting study, Venkatachalam Gopalan et al. performed experimental and numerical analyses on the buckling characteristics of woven flax/the epoxy laminated composite plate under axial compression [8]. In the paper, the authors explored the opportunity of using green composites to substitute oil-based materials. The authors described the case of a plate made of woven flax reinforced with bio epoxy as an alternative to synthetic composites. The results showed that it is possible to obtain a greener solution for structurally demanding uses. Quim Tarrés et al. studied the interface strength and fiber content influence on corn-stover-fiber-reinforced bio-polyethylene composites’ stiffness [9]. The researchers explored the ability of a green composite made of corn fibers as reinforcement to BioPE to replace commodity materials. The authors obtained composite materials using Young’s modulus in line with commercial counterparts. Furthermore, a micromechanics analysis allowed for the establishing of the impact of fiber mean orientation over the composite properties. Ferran Serra-Parareda et al. explored the “Stiffening Potential of Lignocellulosic Fibers in Fully Biobased Composites: The Case of Abaca Strands, Spruce TMP Fibers, Recycled Fibers from ONP, and Barley TMP Fibers” [10]. The authors showed the changes in the stiffness of the composites and their dependence on reinforcement nature and treatment, opening the possibility of choosing a certain reinforcement or treatment based on the envisaged properties of its composites. 

In this Special Issue, we also find works related to the use of natural and/or biodegradable materials such as cellulose versus synthetic polymers. In this regard, the paper by Rudolf Kiefer et al., entitled “Wider Potential Windows of Cellulose Multiwall Carbon Nanotube Fibers Leading to Qualitative Multifunctional Changes in an Organic Electrolyte” [11], explores the use of cellulose combined with electroactive components such as multiwall carbon nanotubes (CNTs) to form composites. These materials can be used as actuators, sensors, and energy storage devices.

There were some articles devoted to the evaluation of the properties of novel materials. Mohsen Bahrami et al. compared the mechanical, thermal, and durability properties of hot-pressed PA11 and PA12 [12]. Both polymers are chemically similar, differing only by one carbon. The results showed that PA12 was more ductile than the brittle PA11. Additionally, water absorption for PA12 and PA11 was measured as being as low as 1.5 and 0.8 wt%, respectively, causing no plasticization and having no impact on dimension or hardness changes. The main parameter that differentiated the materials was the higher crystallinity of PA12, playing a noticeable role in the durability of such a material. Fatin Najwa Joynal Abedin et al. studied the effect of graphene oxide and SEBS-g-MAH compatibilizer on the mechanical and thermal properties of acrylonitrile-butadiene-styrene/talc composite [13]. The authors prepared composite materials with different percentages of the constituents and studied the impact of the phase dosages on the mechanical and thermal properties of the materials. The authors found that talc decreased the stiffness of the composites. Moreover, including graphene oxide with a compatibilizer noticeably improved the thermal and mechanical properties of ABS-based composites. Necar Merah, Farhan Ashraf, and Mian M. Shaukat performed a review on the mechanical and moisture barrier properties of epoxy–nanoclay and hybrid epoxy–nanoclay glass fiber composites [14]. In the review, the authors thoroughly discussed the properties of epoxy clay nanocomposites and proved their superior properties over pristine matrices. The authors conducted a thorough review of the available information on the subject.

Some of the papers researched the properties of composites and searched for greener materials or materials that allow lightweight solutions. Chen Chen, Peng Wang, and Xavier Legrand studied the effect of core architecture on Charpy impact and the compression properties of tufted sandwich structural composites [15]. The authors presented a sandwich structure that was able to replace a polypropylene foam core. Using the proposed sandwich, the compressive strength of materials increased from 45 to 86%, depending on the nature of the substituted core material. Moreover, due to their density, the inclusion of sandwiches noticeably increased the specific properties. Nguyen Quang Khuyen et al. researched the use of laminates of commercially available fabrics for anti-stab body armor [16]. The authors aimed to substitute Kevlar, aiming at increasing the stab-proofing capabilities of body armor. The authors used commercial linen, silk fabrics, and adhesives to prepare the laminates. In addition to the properties of the fabrics, the authors found that adhesives had a noticeable impact on the anti-stab properties of the composites. 

The contributions of the authors to this Special Issue on “Polymer Composite Analysis and Characterization” can be valuable for researchers, engineers, architects, designers, and other practitioners involved in the formulation, characterization, and use of polymer composites. The papers show the importance of novel polymer analysis procedures and the opportunities for further research. 

We would like to express our gratitude to all of the authors that have contributed to the Special Issue and all of the reviewers involved in the process for their generous work. We must thank the *Polymers* editorial board for allowing us to edit this Special Issue, and for their support and patience.
